# ADAM8 inactivation modulates cellular component assembly gene expression in the mouse intervertebral disc

**DOI:** 10.1016/j.gendis.2024.101467

**Published:** 2024-11-19

**Authors:** Ken Chen, Zuozhen Tian, Motomi Enomoto-Iwamoto, Yejia Zhang

**Affiliations:** a Departments of Physical Medicine & Rehabilitation, Perelman School of Medicine, University of Pennsylvania, Philadelphia, PA 19146, USA; b School of Dental Medicine, University of Pennsylvania, Philadelphia, PA 19104, USA; c Department of Orthopedics, Xiangya Hospital, Central South University, Changsha, Hunan 410008, China; d Department of Orthopedics, University of Maryland School of Medicine, Baltimore, MD 21201, USA; e Section of Rehabilitation Medicine, Corporal Michael J. Crescenz Veterans Affairs Medical Center, Philadelphia, PA 19104, USA

Dear Editor

ADAM8 (ADAM metallopeptidase domain 8, aka MS2/CD156/CD156a) is a membrane-anchored proteinase. ADAM8 and its proteolytic product, fibronectin fragments, are elevated in degenerative human intervertebral discs (IVDs).^1^ Pathological fibronectin fragments can trigger further disc degeneration. ADAM8 proteolytic activities and fibronectin fragments may accelerate degenerative disc disease, a common clinical problem leading to tremendous socioeconomic burdens in the United States.

An ADAM8-inactivation mouse model has been generated by introducing a point mutation, replacing the Glutamic acid (E) at position 330 with a Glutamine (Q; abbreviated as *Adam8*^*EQ*^).^2^ The E to Q mutation prevents shedding of the prodomain, thereby preventing self-activation of the enzyme. ADAM8 inactivation resulted in higher proteoglycan content and thicker annulus fibrosus in intact discs.^4^ Despite the morphological differences between the *Adam8*^*EQ*^ and wild type (WT) mouse discs (Fig. 1A), no significant gene expression was detected in the limited panel of markers examined previously.^3^, ^4^ To examine the alteration of biological pathwayss in response to ADAM8 inactivation, an unbiased approach comparing the transcriptome of IVDs in the mutant and WT mice was used. Messenger RNA was isolated from the *Adam8*^*EQ*^ and wild type (WI) mouse IVDs, and bulk RNAseq was performed followed by transcriptomic data analysis. The RNASeq datasets generated and analyzed during this study have been deposited in the NCBI Gene Expression Omnibus (GEO) repository with the accession number GSE255262.

***IVDs in the Adam8***^***EQ***^***mutant mice displayed different gene expression profiles.*** Among the 17,489 genes identified by RNAseq, 94 genes were differentially expressed (*p.adj*<0.05) comparing *Adam8*^*EQ*^ and WT mouse IVDs. The genes were further sorted according to fold changes from high to low, resulting in 25 upregulated and 69 downregulated genes. Four upregulated and 38 downregulated genes with highest fold changes were selected for heatmap, which visualizes clearly different expression profiles between WT and mutant mice (Fig. 1B).

**Figure 1:**
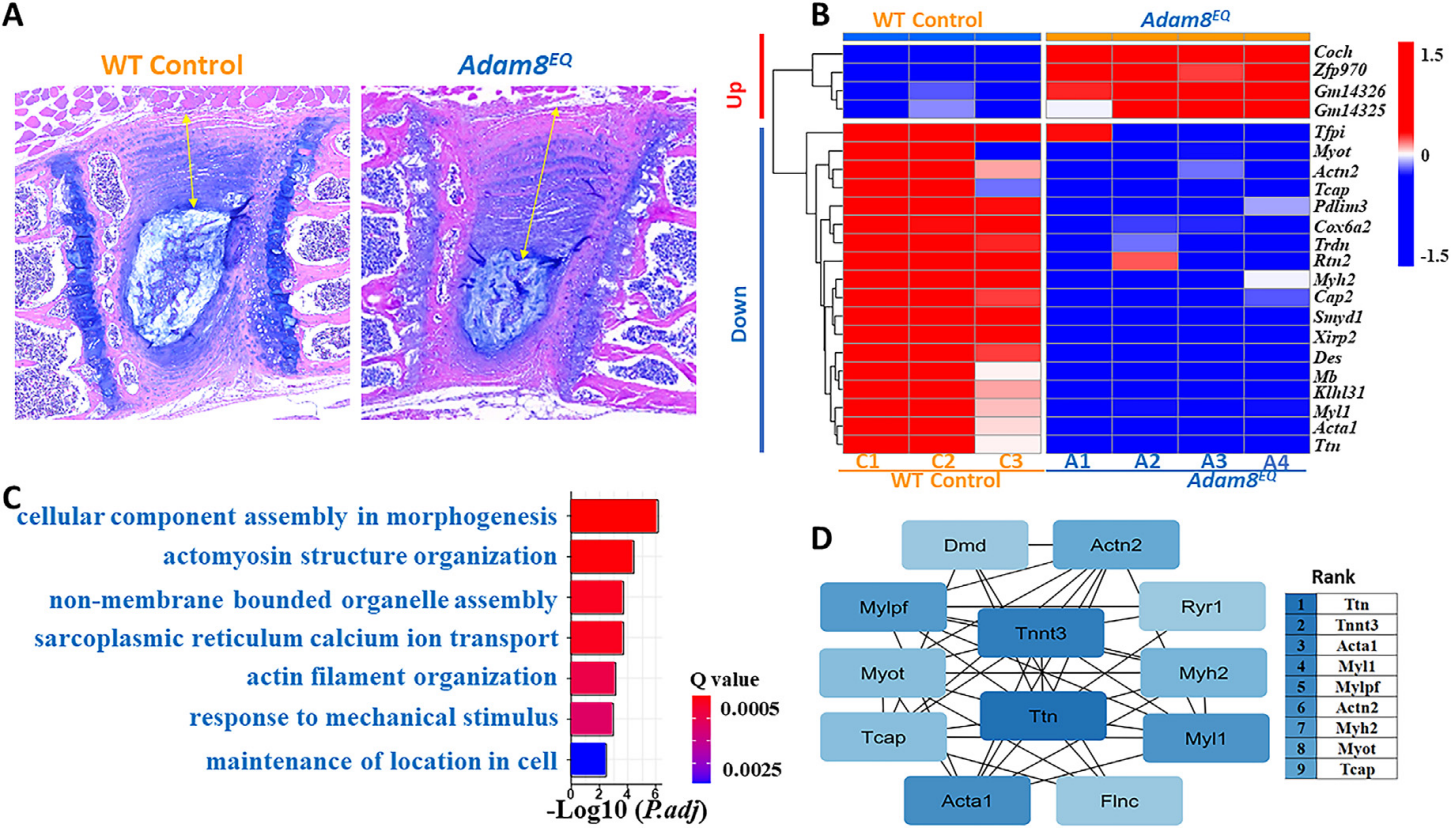
**Differentially Expressed Genes Between Mutant *Adam8***^***EQ***^**and Wild Type (WT) Control Mouse Intervertebral Discs (IVDs). A**: lumbar IVDs stained with Alcian blue; **Arrows:** indicate thickness of the annulus fibrosus; **B: heatmap; e**ach column represents data from one ***Adam8***^***EQ***^ or WT control mouse IVD.**C1-3:** WT controls; **A1-4:** Adam8 mutant; **red**: upregulated genes; **blue**: downregulated genes. **C:** pathway analysis; ***P.adj***: adjusted *p*-value; **Q value:** estimated false discovery rate. **D:** network analysis; **rank:** genes with larger to smaller number of network connections; ***Ttn***: titin; ***Acta*:** actin alpha; ***Myh*:** myosin heavy chain; ***Myl*:** myosin light chain; ***Mylpf*:** Myl phosphorylatable, fast skeletal muscle; ***Actn*:** actinin; ***Myot*:** myotilin; ***Tcap*:** Telethonin.

Cellular component assembly pathway was ***over represented*** among ***genes*** ***differentially*** ***expressed*** ***in Adam8***^***EQ***^ ***and WT control mice****.* A protein list was generated based on fold-changes. The 94 genes with *p.adj*<0.05 were separated into upregulated genes (*Adam8*^*EQ*^/WT ratio>1), and downregulated genes (*Adam8*^*EQ*^/WT ratio<1). Gene Ontology (GO) analysis for biological processes was performed with the R software and various R packages. Cellular component assembly was the most overrepresented pathway among downregulated genes (Fig. 1C). When both up- and downregulated genes were analyzed together, muscle differentiation and myofibril assembly were the two most overrepresented biological pathways among downregulated genes. Histone (H)3 deacetylation was the most overrepresented pathway among the upregulated genes (Fig. S1A).

**Protein–Protein Interaction (PPI) Network Visualization**. The gene list described above was analyzed based on STRING database (https://string-db.org/, Version: 11.5) with the CytoScape software, with minimal interaction score set at high confidence (0.70). Titin (*Ttn*) has the highest network connection among downregulated genes (Fig. 1D). Interestingly, period (per) 1 and 2 genes, encoding proteins known to drive the circadian rhythm, were among the 25 upregulated genes (Fig. S1B).

ADAM8 is known to cleave fibronectin in IVD tissues, generating pathological fibronectin fragments,^1^ which may trigger further disc degeneration. Previous examination of a mouse model revealed that IVD tissues with inactivate ADAM8 retained more proteoglycans and had thicker AF.^4^ In response to injury, the IVD tissues of mutant mice expressed higher levels of type II collagen gene, but also higher levels of inflammatory marker genes including *Cxcl1* and *Il6*.^5^ In these studies, a candidate marker approach was used, where a panel of markers was selected based on past work by our group and others. No statistically significant gene expression changes were detected among the limited panel of genes studied, despite morphological changes in the intact IVDs of ADAM8-inactivation mice compared with WT controls.^3^, ^4^ To elucidate the mechanism of the morphological differences, an unbiased approach was used to compare the entire transcriptome of IVDs in ADAM8-inactivation and WT mice.

Among the 17,489 genes with detectable levels of expression in the mouse IVDs, profound changes in gene expression profiles have been found, with most genes never studied previously in the IVDs (Fig. 1). Cellular component assembly and response to mechanical stimulus pathways were downregulated. We have searched nucleus pulposus cell single cell RNA sequencing data, and found genes with the highest magnitude of changes expressed in various subpopulations of intact NP cells.^1^ Among the genes downregulated in *Adam8*^*EQ*^ mice compared with WT, *Ttn* (Titin), encodes a large protein, and has the largest protein–protein interaction (PPI). *Tfpi* (Tissue Factor Pathway Inhibitor) encodes a protein in the coagulation process. *Zfp970* (Zinc Finger Protein 756) and *Coch* (Cochlin) are among the genes most upregulated in the *Adam8*^*EQ*^ mouse IVDs. *Zfp970* encodes a member of the zinc finger protein family that modulates gene expression, while *Coch* encodes a protein that plays a role in the control of cell shape and motility. None of the above genes highly regulated by ADAM8-inactivation had been well described in IVDs before the current work. Future directions include real-time PCR and immunostaining to confirm the distribution of the genes and proteins. It is also important to be aware that some genes without detectable level of expression in intact IVDs may be induced in pathological states. Another limitation is that young adult male mice on the DBA were used in this study, and genetic background, sex and age are known to influence levels of gene expression.

In summary, an unbiased bulk RNAseq approach was used to compare the transcriptomes of ADAM8-inactivated and WT control mouse IVDs. Profound differences in the gene expression profiles were detected between the mutant and WT mouse IVDs, which could explain their morphological differences reported previously.^4^

## Data availability statement

The RNASeq datasets generated and analyzed during this study have been deposited in the NCBI Gene Expression Omnibus (GEO) repository with the accession number GSE255262. Additional study data will be made available upon reasonable request to the corresponding author.

## CRediT authorship contribution statement

**Ken Chen:** Conceptualization, Data curation, Investigation, Writing – original draft, Writing – review & editing. **Zuozhen Tian:** Conceptualization, Data curation, Writing – original draft, Writing – review & editing. **Motomi Enomoto-Iwamoto:** Investigation, Writing – original draft, Writing – review & editing. **Yejia Zhang:** Conceptualization, Writing – original draft, Writing – review & editing.

## Conflict of interests

There is no financial or non-financial conflicts of interest to report by the authors.

## Figures and Tables

**Figure S1:**
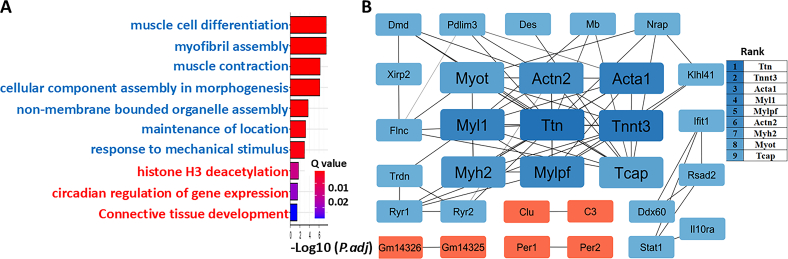
Differentially Expressed Genes Between *Adam8*^***E/Q***^**and Wild Type (WT) Control Mouse Intervertebral Discs (IVDs). A:** pathway analysis; ***P.adj***: adjusted p-value; **Q value:** estimated false discovery rate; **STK**: serine/threonine kinase. **B:** network analysis; **red**: upregulated genes; **blue**: downregulated genes; **rank:** genes with large number of network connections; ***Ttn***: titin; ***Acta*:** actin alpha; ***Myh*:** myosin heavy chain; ***Myl*:** myosin light chain; ***Mylpf*:** Myl phosphorylatable, fast skeletal muscle; ***Actn*:** actinin; ***Myot*:** myotilin; ***Tcap*:** Telethonin.
