# Supplementary material for: ADAM8 inactivation modulates cellular component assembly gene expression in the mouse intervertebral disc
Source: Genes Dis. 2024 Nov 19;12(3):101467. doi: 10.1016/j.gendis.2024.101467 (PMC11804532; doi:10.1016/j.gendis.2024.101467)
Supplement: Multimedia component 1 [file mmc1.docx]

**Supplementary Materials and Methods.**

***Mice, intervertebral disc tissue isolation and RNA extraction.*** A breeding pair of *Adam8^EQ^* mice was gifted by Dr. AnneMarie Malfait. DBA/1LacJ mice (the Jackson Laboratory, Bar Harbor, ME, USA) were bred as WT controls. Mice were housed under pathogen-free conditions with environmental enrichment. A total of 7 mice (4 *Adam8^EQ^* and 3 WT mice), age 10-12 weeks, were used. Tail IVDs were isolated under a dissection microscope, soaked in RNALater, and stored in a -80 ^0^C freezer. Total cellular RNA was extracted by the Trizol method, and purified using a RNeasy Micro Kit (Qiagen).

***RNASeq.*** RNA Library Preparation and Sequencing was performed by Azenta US (South Plainfield, NJ). Specifically, RNA samples were quantified using Qubit 2.0 Fluorometer (ThermoFisher Scientific, Waltham, MA, USA) and RNA integrity was checked with 4200 TapeStation (Agilent Technologies, Palo Alto, CA, USA). rRNA depletion sequencing library was prepared by using QIAGEN FastSelect rRNA HMR Kit (Qiagen, Hilden, Germany). RNA sequencing library preparation uses NEBNext Ultra II RNA Library Preparation Kit for Illumina by following the manufacturer’s recommendations (NEB, Ipswich, MA, USA). Briefly, enriched RNAs were fragmented for 15 minutes at 94°C. First strand and second strand cDNA were subsequently synthesized. cDNA fragments were end-repaired and adenylated at 3’ends, and universal adapters were ligated to cDNA fragments, followed by index addition and library enrichment with limited cycle PCR. Sequencing libraries were validated using the Agilent Tapestation 4200 (Agilent Technologies, Palo Alto, CA, USA), and quantified using Qubit 2.0 Fluorometer (ThermoFisher Scientific, Waltham, MA, USA) as well as by quantitative PCR (KAPA Biosystems, Wilmington, MA, USA). The sequencing libraries were multiplexed and clustered on one flowcell. After clustering, the flowcell was loaded on the Illumina HiSeq instrument according to the manufacturer’s instructions. The samples were sequenced using a 2x150 Pair-End (PE) configuration Raw sequence data (.bcl files) generated from Illumina HiSeq were converted into fastq files and de-multiplexed using Illumina bcl2fastq program version 2.20. One mismatch was allowed for index sequence identification.

**Data Analysis**. After demultiplexing, sequence data were checked for overall quality and yield. Then, sequence reads were trimmed to remove possible adapter sequences and nucleotides with poor quality using Trimmomatic v.0.36. The trimmed reads were mapped to the reference genomes using the STAR aligner v.2.5.2b. The STAR aligner is a splice aware aligner that detects splice junctions and incorporates them to help align the entire read sequences. BAM files were generated as a result of this step. Unique gene hit counts were calculated by using feature counts from the Subread package v.1.5.2. Only unique reads within exon regions were counted. After extraction of gene hit counts, the gene hit counts table was used for downstream differential expression analysis. Using DESeq2, a comparison of gene expression between the groups of samples was performed. The Wald test was used to generate p-values and Log2 fold changes.

**Generation of Heatmap.** 17,489 genes examined by RNASeq had meaningful readings. A differential expressed gene list was generated by limiting *P.adj* to <0.05, resulting in 94 genes in this category. The genes were further sorted according to log2 fold changes from high to low, resulting in 25 upregulated and 69 downregulated genes in the *Adam8^EQ^* mice compared with WT controls. For upregulated genes, we selected 4 genes based on their highest rankings. For downregulated genes, we selected the 38 genes with highest fold changes comparing WT with *Adam8^EQ^* mice. After inputting the chosen upregulated genes and downregulated genes, a heatmap was generated using R package pheatmap, in which rows (which represent gene expression) were scaled and hierarchically clustered, and a gap was inserted among columns to differentiate the intact control and injured groups.

**Gene ontology (GO).** A protein list was generated based on fold-changes. The 94 genes with *p.adj*<0.05 were separated into upregulated genes (*Adam8^EQ^*/WT ratio>1), and downregulated genes (*Adam8^EQ^*/WT ratio<1). GO analysis for biological processes was performed with the R software and various R packages.

**Protein-Protein Interaction (PPI) Network Visualization**. Ranking PPI networks were downloaded from STRING database (https://string-db.org/, Version: 11.5) and imported into CytoScape software (Version: 3.9.1). The minimal interaction score was set at high confidence (0.70). Confidence scores are scaled between 0 and 1, with 1 corresponding to the estimated likelihood of a given association being true. Disconnected nodes in the network were not displayed. CytoHubba plugin was used to rank nodes with the MCC method. The parameters of nodes and edges were adjusted based on node rankings and the combined interaction scores predicted by STRING database, thus visualizing PPI networks in a more intuitive and clear way.
